# Evaluation of equation as a classifier for kidney function in domestic cats: a proof-of-concept study

**DOI:** 10.1038/s41514-026-00417-2

**Published:** 2026-05-25

**Authors:** Qinghong Li, Linxing Yao

**Affiliations:** 1 Nestlé Research, Saint Louis, MO USA; 2 College of Veterinary Medicine, Auburn University, Auburn, AL USA; 3 Analytical Resources Core - Bioanalysis and Omics, Colorado State University, Fort Collins, CO USA

**Keywords:** Diseases, Medical research, Nephrology

## Abstract

In human medicine, estimated glomerular filtration rate (eGFR) is routinely used to assess renal function. In cats, reliable eGFR formulas are not yet available. To test the hypothesis that a human eGFR equation with four metabolites can effectively distinguish cats with different renal functions, two cohorts of cats were retrospectively enrolled. Cohort 1 included 34 colony cats and cohort 2 had 29 client-owned cats. The metabolite markers were quantified in cat serum and renal function was estimated using the adapted equation (eRF_fel_). Receiver Operating Characteristic Area Under the Curve for eRF_fel_ as a binary classifier were 0.9714 and 0.9958, and 0.9731 and 0.9643 as a ternary classifier for cohort 1 and cohort 2, respectively. In cohort 1, eRF_fel_ was 1.30 ± 0.04 (Mean ± SEM, mL/min/kg), 0.78 ± 0.06, and 0.36 ± 0.05 for healthy control, IRIS Chronic Kidney Disease (CKD) stages 1 and 2 (CKD1/2), and stages 3 and 4 (CKD3/4), respectively, and 1.30 ± 0.06, 0.72 ± 0.05, and 0.47 ± 0.04 for healthy control, CKD stage 2, and CKD stage 3 in cohort 2, respectively (both *P* < 0.0001). Although the original equation was trained on human data, eRF_fel_ was associated positively with urine specific gravity, but negatively with serum symmetric dimethylarginine and creatinine in cats (all *P* < 0.0001). The preliminary results demonstrated the effectiveness of eRF_fel_ as a classifier for feline renal function.

## Introduction

Chronic kidney disease (CKD) is a common disease of cats, with a marked increase in prevalence in older cats aged 12 years or older, and is reported to be the most common cause of death in cats over five years of age^1,2^. Typically, an initial diagnosis of CKD is made using blood and urine markers (serum creatinine, serum symmetric dimethylarginine [SDMA], urine specific gravity [USG]), followed by staging in accordance with International Renal Interest Society (IRIS) guidelines^3,4^. In IRIS stage 1 CKD, cats are non-azotemic, with many cats showing no noticeable clinical signs despite already experiencing a significant loss of nephron function^4,5^. The transition from IRIS stage 2 to stage 4 CKD in cats is characterized by a progressive decline in kidney function, with increasing levels of azotemia and associated clinical signs. While stage 2 typically presents mild or asymptomatic azotemia, stage 4 refers to severe azotemia and is considered end-stage renal failure^4,5^. Indeed, approximately 75% of functioning nephrons need to be lost before serum creatinine levels rise above the reference interval^6^. Serum creatinine levels are also influenced by non-renal factors, such as lean body mass and body weight^7^. Hall et al. reported that serum SDMA increased earlier than creatinine^6^. Brans et al. were unable to confirm the superiority of SDMA to creatinine as a reliable marker for glomerular filtration rate (GFR).

Although GFR measurement is regarded as the gold standard for assessing renal function, it currently involves the use of an intravenous clearance marker, followed by multiple samplings, a process that is stressful for both pets and their owners^8–10^. Thus, additional diagnostic markers and tools that can aid in the early detection and monitoring of kidney dysfunction are crucial for effective management of feline CKD.

In human medicine, estimated GFR (eGFR) measurements are routinely made to assess renal function, as an alternative to using exogenous markers to measure GFR directly. These eGFR assessments are often made using equations based on concentrations of serum creatinine, cystatin C or a combination of both^11^. Unfortunately, exploration of similar creatinine-based eGFR formulas for cats, have not been shown to be reliable^12^. Over recent years, many human studies have evaluated metabolomic approaches to the investigation of CKD both to understand its pathogenesis better and also to find alternative assays that will improve the existing eGFR assays, which still show bias^11,13–21^. Among these, Freed and colleagues^21^ developed a human machine learning model for eGFR using measurements of pseudouridine (Ψ), phenylacetylglutamine (PAGln), N-acetylthreonine (AcThr), and tryptophan (Trp), and a large dataset of CKD patients. The authors demonstrated their assay was more accurate than the eGFR assays based on either creatinine or cystatin C alone. Pseudouridine (Ψ) is synthesized through isomerization of RNA uridine residues^22,23^, and has been reported to be a biomarker for kidney function and CKD progression in humans^13–18^. PAGln and AcThr are derivatives of the amino acid phenylalanine and threonine, respectively, with PAGln recognized as a gut microbiota-produced uremic toxin associated with the risk of cardiovascular diseases, cerebrovascular disorders, and CKD in humans^24–27^. Studies have also associated AcThr, Ψ and C-glycosylated Trp with renal function decline and diabetes^19,20^.

Standard methods for quantification of circulating Ψ, PAGln, and AcThr have been developed in humans^21,26^, but were not so far been applied to cats. As a proof-of-concept study, we wanted to apply these assays, along with a modification of the eGFR equation derived for their use by Freed and colleagues^21^, to see if their measurement in feline serum would be able to distinguish between healthy cats, cats with early and late stages CKD. We hypothesized that the adapted model would effectively discriminate between cats with different levels of renal function and that based on this, further investigations would be warranted.

## Results

### Physical and clinical characteristics of cats

Cats in cohort 1 were all domestic shorthair from the same colony. The mean age of the healthy control group was younger than that of IRIS stage 1 and stage 2 CKD (CKD1/2, *P* = 0.002), while the mean body weight of the healthy control group was greater than those of both CKD1/2 and CKD3/4 (IRIS stage 3 and 4 CKD) group. (*P* = 0.001) (Table 1). No significant association was observed between sex and CKD group (*P* = 0.42).

**Table 1:** Physical and clinical descriptions of cats in cohort 1

	Healthy	CKD1/2	CKD3/4	P value
Sample size	14	14	6	n/a
Age (years)	10.9 ± 0.7^a^	15.4 ± 0.7^b^	11.7 ± 0.6^ab^	0.002
Sex (F/M)	9/5	7/7	5/1	0.42
Weight (kilograms)	4.7 ± 0.3^a^	3.6 ± 0.2^b^	2.9 ± 0.3^b^	0.001
Breed				
DSH	14	14	6	n/a
Clinical measurements				
Serum creatinine (mg/dL)	1.36 ± 0.06^a^	1.69 ± 0.11^a^	3.80 ± 0.50^b^	<0.001
Serum SDMA (μg/dL)	11.0 ± 0.6^a^	17.0 ± 1.0^b^	31.0 ± 2.4^c^	<0.001
USG^*^	1.050 ± 0.002^a^	1.015 ± 0.002^b^	1.011 ± 0.001^b^	<0.001
UPC^*^	0.33 ± 0.19^a^	0.35 ± 0.06^a^	2.35 ± 1.14^b^	0.002

CKD1/2, IRIS stages 1 (*n* = 2) and 2 (*n* = 12) CKD; CKD3/4, IRIS stages 3 (*n* = 4) and 4 (*n* = 2) CKD. DSH, domestic short-haired; SDMA, symmetric dimethylarginine; UPC, urine protein to creatinine ratio; USG, urine specific gravity. P values were obtained from ANOVA for continuous variables or Fisher’s Exact Test for categorial variables. Different superscripted letters denote statistical difference between groups. Age, weight, and clinical variables are reported as mean ± SEM. All cats were neutered.

^*^Urine sample from one CKD3/4 cat was not collected.

Cats in cohort 2 were client-owned. The mean age of the healthy control group was younger that those of IRIS stage 2 CKD (CKD2) and IRIS stage 3 CKD (CKD3) (*P* = 0.003) (Table 2). No difference in body weight or sex was observed between groups (*P* = 0.15 and *P* = 0.47, respectively).

**Table 2:** Physical and clinical descriptions of cats in cohort 2

	Healthy	CKD2	CKD3	P value
Sample size	9	10	10	n/a
Age (years)	13.0 ± 0.8^a^	17.7 ± 0.6^b^	16.2 ± 1.1^b^	0.003
Sex (F/M)	6/3	4/6	4/6	0.47
Weight (kilograms)	3.93 ± 0.23	3.38 ± 0.15	3.84 ± 0.23	0.15
Breed				
DSH	9	6	7	n/a
DLH	0	2	1	n/a
Birman	0	1	0	n/a
Devon Rex	0	1	0	n/a
BSH	0	0	1	n/a
Tiffany	0	0	1	n/a
Clinical measurements				
Serum creatinine (mg/dL)	1.27 ± 0.06^a^	1.93 ± 0.08^b^	3.56 ± 0.23^c^	<0.001
Serum SDMA (μg/dL)	10.8 ± 0.8^a^	17.9 ± 1.5^b^	21.3 ± 0.9^b^	<0.001
USG	1.045 ± 0^a^	1.018 ± 0^b^	1.015 ± 0^b^	<0.001
UPC	0.15 ± 0.02	0.36 ± 0.10	0.48 ± 0.33	0.91

CKD2, IRIS stage 2; CKD3, IRIS stage 3. DSH, domestic short-haired; DLH, domestic long-haired; BSH, British short-haired. SDMA, symmetric dimethylarginine; UPC, urine protein to creatinine ratio; USG, urine specific gravity. *P* values were obtained from ANOVA for continuous variables or Fisher’s Exact Test for categorial variables. Different superscripted letters denote statistical difference between groups. Age, weight, and clinical variables are reported as mean ± SEM. All cats were neutered.

### Quantification of metabolite markers

**Fig. 1:**
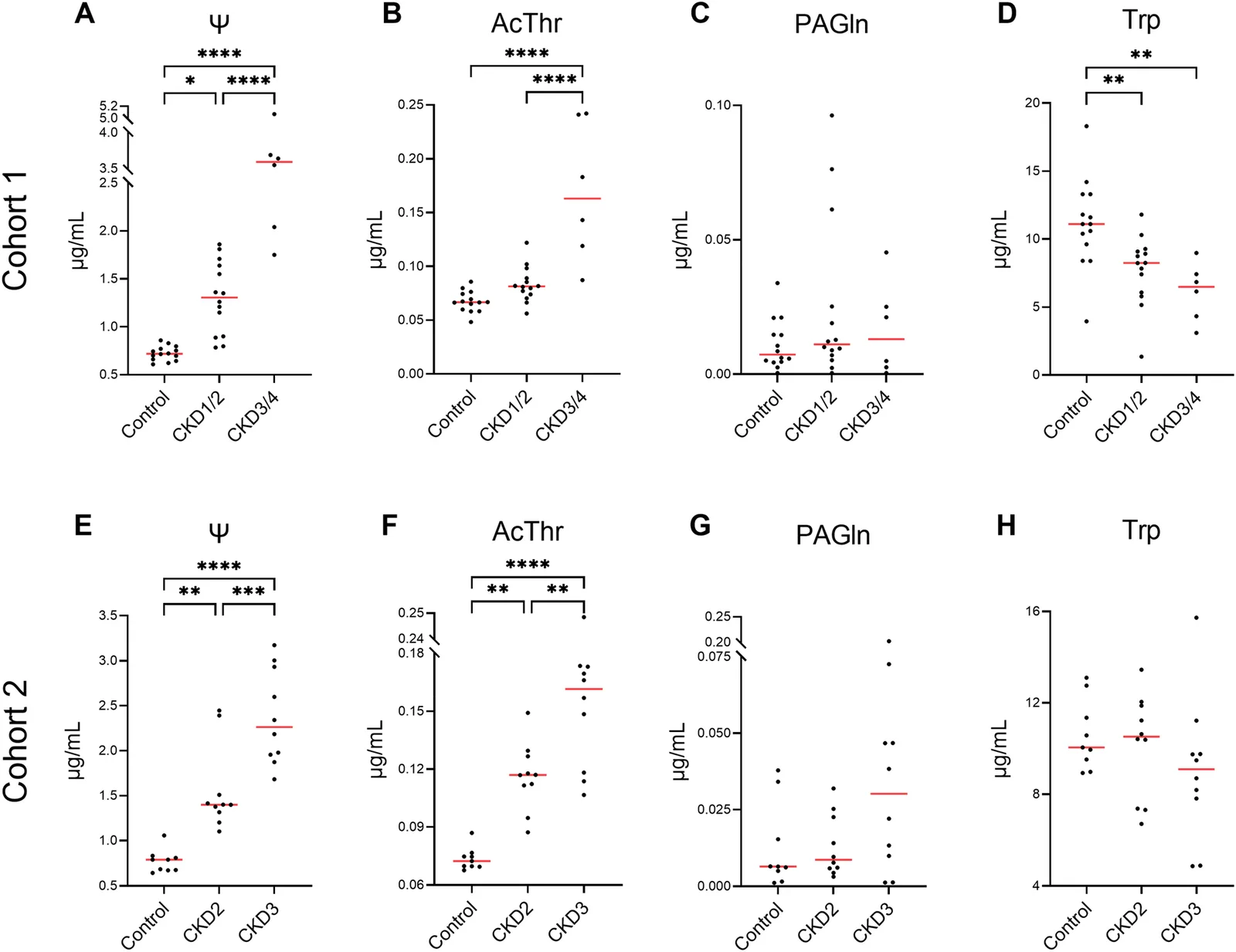
Quantifications of metabolites. The concentrations of pseudouridine (Ψ), acetylthreonine (AcThr), phenylacetylglutamine (PAGln), and tryptophan (Trp) in cohort 1 **A-D**, and cohort 2 **E-H**, respectively. The horizontal red lines represent the medians. The means among groups were compared using ANOVA test followed by Tukey’s HSD multiple comparisons. *P < 0.05; **P < 0.01; ***P < 0.001; ****P < 0.0001. CKD1/2, IRIS stage 1 and stage 2 CKD; CKD3/4, IRIS stage 3 and stage 4 CKD.

In cohort 1, the median (IQR) concentrations of Ψ (μg/mL) for control, CKD1/2, and CKD3/4 groups were 0.72 (0.66, 0.78), 1.31 (0.90, 1.66), and 3.60 (1.97, 4.04), respectively (*P* < 0.0001, Fig. 1A). Similarly, the median concentrations of AcThr (μg/mL) for control, CKD1/2, and CKD3/4 groups were 0.07 (0.06, 0.07), 0.08 (0.07, 0.09), and 0.16 (0.11, 0.24), respectively (*P* < 0.0001, Fig. 1B). No difference in PAGln between groups was observed (*P* = 0.25, Fig. 1C). To the contrary, the concentration of Trp was decreased in CKD1/2 and CKD3/4 compared to the control (*P* = 0.0011, Fig. 1D).

Similar observations were made in cohort 2. The median concentrations of Ψ (μg/mL) for control, CKD2 and CKD3 were 0.79 (1.29, 1.73), 1.40 (1.29, 1.73), and 2.26 (1.93, 2.95), respectively (*P* < 0.0001, Fig. 1E). The levels of AcThr were also higher in both CKD groups compared to the control group (*P* < 0.0001, Fig. 1F). No difference in PAGln or Trp between groups was observed (*P* = 0.09, *P* = 0.37 respectively, Fig. 1G-H).

The concentrations of Ψ, AcThr, PAGln, Trp, and the estimated renal function (eRF_fel_ as defined in the Method) of the healthy non-CKD cats in cohort 1 were not different from those in cohort 2 (Supplemental Table S1).

### Performance of eRF_fel_ as a classifier

**Fig. 2:**
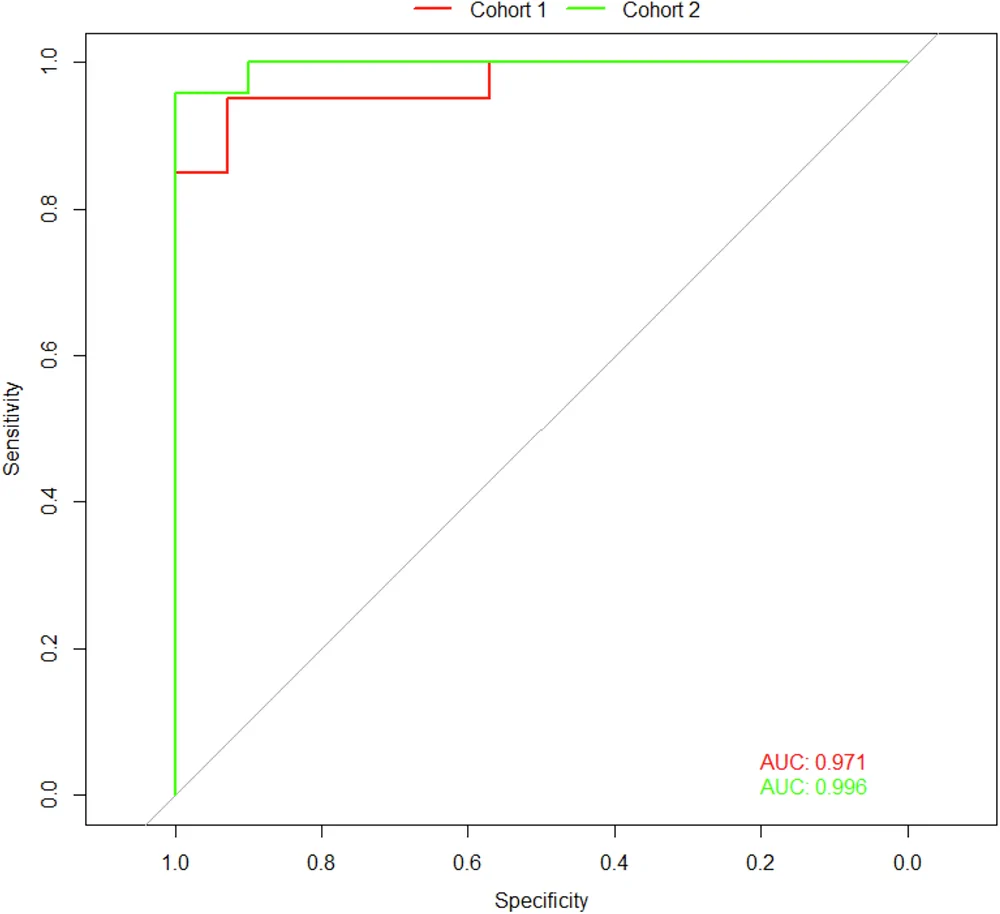
Receiver Operating Characteristic plot and Area Under the Curve. Receiver Operating Characteristic (ROC) curves were generated for cohort 1 (red) and cohort 2 (green). A ROC curve is a graphical representation of true positive rate (sensitivity) against the false positive rate (1-specificity) at various thresholds. The Area Under the Curve (AUC) of a ROC curve is a metric used to evaluate the performance of a machine learning model. An AUC of 1.0 indicates perfect classifications, while an AUC of 0.5 (grey diagonal line) indicates no discrimination. A value above 0.9 is considered excellent.

The overall effectiveness of eRF_fel_ as a binary classifier to correctly separate cats as either being healthy or CKD was assessed using the Receiver Operating Characteristic (ROC) Area Under the Curve (AUC) analysis. The ROC AUC values were 0.9714 (95% CI: 0.9235-1) for cohort 1 and 0.9958 (0.9843-1) for cohort 2 (Fig. 2).

Further, the overall performances of eRF_fel_ as a ternary classifier to correctly classify cats in cohort 1 (healthy, CKD1/2, and CKD3/4) and in cohort 2 (healthy, CKD2, and CKD3) were 0.9731 and 0.9643, respectively.

### Values obtained by eRF_fel_

**Fig. 3:**
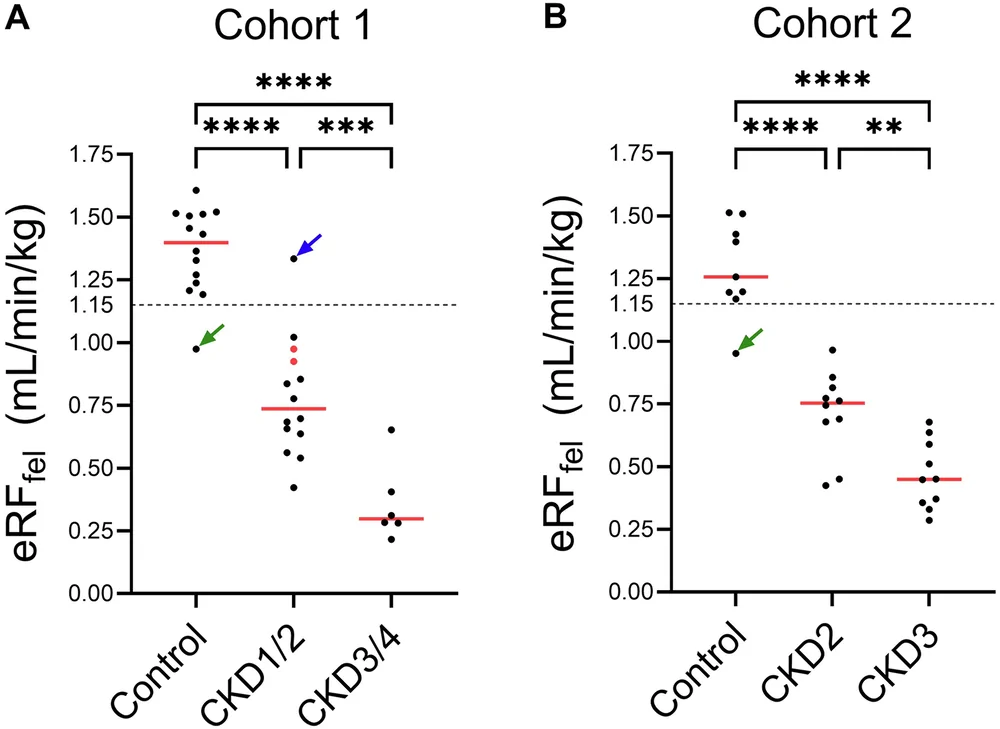
Values obtained by eRF_fel_. The eRF_fel_ values of **A** healthy control, CKD1/2, and CKD3/4 groups in cohort 1 and **B** healthy control, CKD2, and CKD3 groups in cohort 2. The horizontal red lines in the boxplots represent the medians. The two IRIS stage 1 CKD cats the CKD1/2 group of cohort 1 are labeled in solid red circles. The green arrows in **A, B** indicate healthy cats whose eRF_fel_ values were outside the reference range (A, creatinine=1.34 mg/dL and SDMA = 16 μg/dL; **B**, creatinine=1.43 mg/dL and SDMA = 12 μg/dL). The blue arrow in **A** indicates an IRIS stage 2 CKD cat whose eRF_fel_ value was within the reference range (creatinine=1.61 mg/dL and SDMA = 21 ug/dL). The greyish dotted line, y = 1.15 mL/min/kg represents the lower limit of the reference range proposed by the Veterinary Diagnostic Laboratory at Michigan State University. Group means were compared using the ANOVA test followed by Tukey’s HSD multiple comparisons. *P < 0.05, **P < 0.01; ***P < 0.001; ****P < 0.0001.

In cohort 1, the median eRF_fel_ values (mL/min/kg) of the control, CKD1/2, and CKD3/4 groups were 1.34 (1.23, 1.51), 0.74 (0.62, 0.94), and 0.30 (0.26, 0.47), respectively (*P* < 0.0001, Fig. 3A). The eRF_fel_ values of all CKD cats were < 1.15 (below the reference interval for feline GFR) except for one, whose eRF_fel_ was 1.33 (Fig. 3A, blue arrow). The eRF_fel_ values of all healthy cats were > 1.15 except one, whose eRF_fel_ was 0.97 (Fig. 3A, green arrow). The median eRF_fel_ value of the CKD1/2 group was greater than that of CKD3/4 group (*P* < 0.001). Notably, the eRF_fel_ values of the two cats with IRIS stage 1 CKD were 0.97 and 0.93 (Fig. 3A, red circles).

For client-owned cats in cohort 2, the median values of eRF_fel_ (mL/min/kg) for the control, CKD2, and CKD3 groups were 1.3 (1.2, 1.5), 0.75 (0.62, 0.83), and 0.45 (0.35, 0.60), respectively (*P* < 0.0001) (Fig. 3B). The eRF_fel_ of healthy cats were within the reference ranges for feline GFR ( > 1.15 mL/min/kg) except for one, whose eRF_fel_ was 0.95 (Fig. 3B, green arrow).

### Association between eRF_fel_ and physical and clinical features

In cohort 1, eRF_fel_ showed negative correlations with SDMA and creatinine (Spearman correlation coefficient *ρ* = -0.85 and -0.68 respectively, both *P* < 0.0001), but a positive one with USG (*ρ* = 0.82, *P* < 0.0001) (Supplemental Fig. S1A, Supplemental Fig. S2A-C). Similarly, significant associations between eRF_fel_ and SDMA, creatinine, and USG were observed in cohort 2 (*ρ* = -0.81, -0.89, and 0.75, respectively, all *P* < 0.0001) (Supplemental Fig. S1B, Supplemental Fig. S2D-F).

In cohort 1, eRF_fel_ values were negatively associated with age (Spearman *ρ* = -0.38, *P* = 0.028) and positively associated with weight (*P* < 0.0001, *ρ* = 0.67) (Supplemental Fig. S1A). In cohort 2, a negative correlation was observed between eRF_fel_ and age (*ρ* = -0.56, *P* = 0.002), but there was no association with body weight (Supplemental Fig. S1B).

## Discussion

We applied a validated machine learning model developed to estimate GFR in humans^21^ to two cohorts of cats, by introducing a “feline factor” to account for species-specific differences in kidney functions, the differences in measurement units and reference ranges between humans and domestic cats. The adapted equation (eRF_fel_), which uses four metabolite markers as predictor variables, was used to assess renal function in cats. But from our current data, it cannot be assumed to provide an estimate of feline GFR.

The two cohorts of cats, one from a research colony and the other consisting of client-owned cats, were retrospectively enrolled using stored serum samples to assess eRF_fel_ equation’s performance as a classification model to differentiate between healthy cats and cats with CKD and between cats with different severities of CKD. Our results confirmed our hypothesis and demonstrated the potential for using these metabolites in a classification model in cats. While the results of our study provide evidence to support the potential value of assaying these metabolites in the diagnosis and staging of feline CKD, larger prospective studies incorporating directly measured GFR for comparison will be required to fully assess their potential and to establish whether they can be used to estimate GFR in cats. Longitudinal data on healthy senior cats and those suspected of early-stage (i.e. IRIS stage 1 and early stage 2) CKD, will also enhance our understanding of these metabolite assays. Wider metabolomic studies in such cats may also yield other metabolite markers that may be of interest in feline CKD, as has been shown in humans^14–19^.

One of the key differences between the model used by Freed and colleagues and previous ones is that it does not depend on creatinine levels^21^. Although creatinine is widely recognized as a standard surrogate marker for renal function, its levels remain within the normal interval until approximately 75% of nephrons are nonfunctioning^6^. In addition, creatinine production can be influenced by non-renal factors such as lean muscle mass. Pseudouridine (Ψ), a nucleoside whose levels are not dependent on muscle mass, has been implicated as a biomarker for kidney function and CKD progression^13–18^. In cats, elevated blood Ψ levels have also been reported in CKD, and more broadly, in kidney diseases^28^. Pseudouridine is the most abundant posttranscriptional modifications of cellular RNAs, and is produced via Ψ synthases through isomerization of uridine residues that are already part of an RNA chain^22,23^. Although Ψ was discovered more than a century ago, its enzymatic mechanism and function of this modification are still not fully elucidated. Phenylacetylglutamine and AcThr are both amino acid derivatives. PAGln is gut microbiota-derived uremic toxin that has been associated with the risk of cardiovascular diseases, cerebrovascular disorders, and CKD in humans^24–27^. Studies have associated AcThr, Ψ and C-glycosylated tryptophan (Trp) with renal function decline in human patients with type 1 diabetes with impaired renal function^19^ and in patients with diabetic kidney disease^20^. To date, the absolute concentrations of Ψ, AcThr, or PAGln in the circulation of cats have not been reported. Trp, an essential amino acid for cats, is primarily metabolized through three pathways: the indole pathway, the serotonin pathway, and the predominant kynurenine pathway. Our study suggests that post-transcriptional and post-translational alterations of these metabolites may potentially contribute to the pathophysiology of CKD in cats. One of the exciting potential benefits of the metabolomic approach to exploring markers of CKD is that they may yield both more accurate data for eGFR, but also may provide better diagnostics for the advancement of CKD, as some metabolites may infer a greater risk of progressive disease^13–15,18,25,26,29^.

The reference interval for serum iohexol clearance for cats provided by the Veterinary Diagnostic Laboratory at Michigan State University (MSU) is 1.15-2.73 mL/min/kg. Heiene et al. showed a slightly wider reference range of 1.0-3.5 mL/min/kg for iohexol in 51 clinically normal, non-azotemic cats^8^. The eRF_fel_ values of 93% (13/14) of the healthy cats in cohort 1 and 89% (8/9) healthy cats in cohort 2 fell within the reference interval for eGFR by MSU, while eRF_fel_ of 95% (20/21) of the CKD cats in cohort 1 and all CKD cats in cohort 2 were below the interval. Significantly, the eRF_fel_ values of the two IRIS stage 1 CKD cats in cohort 1 were close to the lower threshold of the normal ranges proposed by MSU and Heiene and colleagues. Because cats in the study were retrospectively enrolled, measured GFRs were not available. An important next step would be to understand the accuracy of this eRF_fel_ equation by comparing it with measured GFR. Additionally, evidence of the ability to predict renal function decline in comparison to current markers will strengthen its potential for future clinical uses.

There were some important differences between the two cohorts of cats used in this study. The cats in cohort 1 lived in the same environment in North America, whilst cats in cohort 2 lived in different homes in Europe with different environmental variables. In cohort 1, cats with CKD had significant increases in UPC, while no difference in UPC was observed in cohort 2. Similar to human studies, we were unable to rule out cats with subclinical conditions, which might confound our classification of the cats. Fasting blood samples were collected in cohort 1, but not cohort 2. Diet composition is considered as one of the key factors that influence the circulating metabolome in humans^30^ and pets^31^. These differences in environment and fasting conditions could potentially contribute to the variations found in the measured metabolites. Diet and medications are other potential confounding factors. Cats with CKD are usually on special renal diets designed to support the kidney function. Many CKD cats experience inappetence and body weight loss. So dry or wet or both diet types are offered to encourage food consumptions. Moreover, some CKD cats were on medications to regulate high BP or suppress hyperthyroidism.

We further explored the relationships between eRF_fel_ and some key physical and clinical features associated with feline CKD. Although the equation was trained on human data and the algorithm did not have any feline-related knowledge, the eRF_fel_ showed a strong correlation with creatinine, SDMA, and USG. Additionally, the equation was associated to a lesser extent with age and body weight. We believe that the association with these physical, biochemical and clinical features is a reflection of the health status of these cats. For example, weight was not significantly different between the groups in cohort 2, and similarly its correlation with eRF_fel_ became insignificant. Hence, we considered the observation as an encouraging sign, rather than as a source of concern for confounding effects. Nevertheless, the performance of this equation as an estimator for feline renal function and against the current renal markers such as creatinine have yet to be determined.

In conclusion, this study has demonstrated that evaluating the combination of four metabolites previously shown to have collectively provided a good assessment of eGFR in humans, was able to provide good distinction between healthy cats and cats with different stages of CKD. This strongly suggests that further studies should be undertaken to evaluate these metabolites as potential markers for eGFR in cats and potential markers of progressive disease in this species.

## Methods

### Prediction model for feline renal function

A multivariate machine learning model developed and trained for human eGFR^21^ was modified and adapted for cats. The model depends on four metabolite markers, Ψ, AcThr, PAGln, and Trp, as predictor variables.

The reference ranges for serum iohexol clearance for cats provided by Michigan State University Veterinary Diagnostic Laboratory (https://cvm.msu.edu/vdl) are 1.15-2.73 mL/min/kg. In humans, a GFR of 90 mL/min/1.73 m^2^ or higher is considered normal according to the guidelines from the National Kidney Foundation. A “feline factor” (*f*) was therefore introduced, based on these GFR values, to modify the original equation for use in cats.

Original eGFR equation reported by Freed and colleagues^21^:

$${\rm{eGFRmet}}=28.106\times {\Psi }^{-0.805}\times {{\rm{PAGln}}}^{-0.047}\times {{\rm{AcThr}}}^{-0.075}\times {{\rm{Trp}}}^{0.266}$$

New estimated feline renal function (eRF_fel_) formula used here:

${\rm{eRFfel}}=f\times (28.106\times {\Psi }^{-0.805}\times {{\rm{PAGln}}}^{-0.047}\times {{\rm{AcThr}}}^{-0.075}\times {{\rm{Trp}}}^{0.266})$ Where the “feline factor” f is a constant, $f=\frac{1.15}{90}=0.0128$ m^2^/kg, and Ψ, PAGln, AcThr, and Trp represent β-pseudouridine, phenylacetylglutamine, N-acetylthreonine, and tryptophan, respectively. All metabolites are expressed in μg/mL.

### Animals

In this study, cohort 1 were colony cats, and cohort 2 were client-owned cats, both being enrolled retrospectively^32,33^. In cohort 1, there were 34 colony cats, all domestic short-hair, including 14 healthy non-CKD cats (control group), 14 with early-stage CKD (CKD1/2 group, including 2 IRIS stage 1 and 12 IRIS stage 2 cats), and 6 with late-stage CKD (CKD3/4 group, including 4 IRIS stage 3 and 2 IRIS stage 4) (Table 1). Cohort 2 consisted of 29 client-owned cats, including 9 healthy control cats, 10 IRIS stage 2 (CKD2) and 10 IRIS stage 3 cats (CKD3) (Table 2). All cats were neutered.

All cats received a complete physical examination with a body condition score^34^, muscle condition score^35^, medical history evaluation, complete blood count, serum biochemistry profile including SDMA, indirect systolic blood pressure (sBP), and urinalysis including urine protein to creatinine ratio (UPC). A cat was diagnosed with CKD if USG < 1.035, and had elevated serum creatinine or SDMA levels on at least two different assessments, where the medical history, and physical examination findings supported the diagnosis. Cats diagnosed with CKD with regulated hyperthyroidism and hypertension were allowed to enroll. Cats diagnosed with CKD were then staged using the IRIS guidelines. A cat was assigned IRIS stage 1 if its serum creatinine concentrations was <1.6 mg/dL, otherwise IRIS stages 2-4 as appropriate. A cat was considered healthy if the following criteria were met: (1) USG ≥ 1.035, (2) serum SDMA < 18 μg/dL, (3) serum creatinine <1.6 mg/dL, (4) sBP <160 mmHg, and (5) no clinical evidence of kidney disease, urinary tract infection or any other major systemic diseases such as cancer, heart disease, or diabetes mellitus at the time of examination. Cats within each cohort were evaluated by the same board-certified veterinary internists. The study protocol was reviewed and approved by the Institutional Animal Care and Use Committee of the Nestlé Purina PetCare Company with the approval number 7427. An owner consent was obtained for all client-owned cats.

### Sample collection

Overnight fasting blood samples were collected from the colony cats that lived in North America (cohort 1). Client-owned cats that lived in Europe (cohort 2) were not fasting at the time of blood draw. Serum samples were immediately stored at -80°C until use.

### LC/MS quantification of metabolites

Cat serum samples were analyzed by liquid chromatography-tandem mass spectrometry (LC-MS/MS) method. Serum metabolites of cohort 1 were quantified by a commercial laboratory (Metabolon Inc., Morrisville, NC). Serum samples from cohort 2 were quantified at the Analytical Resources Core at Colorado State University (Fort Collins, CO), and the detailed protocol was described below.

Reference standards N-acetyl-L-threonine was purchased from Santa Cruz Biotechnology (Dallas TX), β-pseudouridine and phenylacetyl L-glutamine were from Cayman Chemical (Ann Arbor, MI), tryptophan was from Sigma-Aldrich (St.Louis, MO), phenylacetyle-d_5_-L-glutamine and β-pseudouridine-^13^C,^15^N_2_ were from LGC (Manchester, NH), N-acetyl-d_3_-L-threonine-2,3-d_2_ was from CDN Isotopes (Quebec, Canada), tryptophan-^13^C_11_ was from Cambridge Isotopes (Tewksbury, MA). All solvents mentioned elsewhere in section 4.4. were purchased from Fisher Scientific (Hampton, NH). To extract the metabolites, serum samples (50 μL) were mixed with ice-cold methanol (200 μL) and internal standard mix (10 μL containing 600 ng/mL of N-acetyl-d_3_-L-threonine-2,3-d_2_, 80 ng/mL of phenylacetyle-d_5_-L-glutamine, 3000 ng/mL of β-pseudouridine-^13^C,^15^N_2_, 400 ng/mL of tryptophan-^13^C_11_ in 50% methanol/water). The mixture was vortexed for 3 s, incubated at -20°C overnight, followed by centrifugation at 15,000 *g* x 10 min and 4°C. The supernatant was analyzed on a Waters ACQUITY Classic UPLC coupled to a Waters Xevo TQ-S triple quadrupole mass spectrometer (Milford, MA). A quality control (QC) sample that was pooled from study samples was injected after every six study samples. Chromatographic separations were carried out on a Waters Atlantis Premier BEH Z-HILIC VanGuard FIT column (2.1 ×100 mm, 1.7 μM). Mobile phases were (A) 10 mM ammonium formate in water with 0.1% formic acid and (B) acetonitrile with 0.1% formic acid. The LC gradient was as follows: time=0 min, 80% B; time=0.5 min, 80% B; time=1.6 min, 35% B; time=1.61 min, 5% B; time=3.61 min, 5% B; time=3.62 min, 80% B; time=6 min, 80% B. Flow rate was 0.4 mL/min and injection volume was 2 μL. Samples were held at 6°C in the autosampler, and the column was operated at 40°C. Mass detector was operated in ESI positive mode with the capillary voltage set to 0.7 kV. Inter-channel delay was set to 3 msec. Source temperature was 150°C and desolvation gas (nitrogen) temperature 500°C. Desolvation gas flow was 1000 L/h, cone gas flow was 150 L/h, and collision gas (argon) flow was 0.25 mL/min. Nebulizer pressure (nitrogen) was set to 7 Bar. The MS acquisition functions were scheduled by retention times. The retention time, multiple reaction monitoring (MRM) transitions, dwell time, cone energy and collision energy of each compound were described in Supplemental Table S2. Data were processed using Skyline open source software^36^. Peak areas of target compounds detected in biological samples were normalized to the peak areas of the corresponding internal standard. The linear fit of calibration curve was used to extrapolate the concentrations of target compounds.

### Receiver Operating Characteristic (ROC) Area Under the Curve (AUC)

The overall performance of the eRF_fel_ as a binary classifier to discriminate between healthy cats and cats with CKD and a ternary classifier to distinguish among three classes (e.g. healthy non-CKD, CKD1/2, and CKD3/4) were assessed using ROC curves. The ternary AUC is defined as the mean of several pairwise AUC scores using the Hand and Till method^37^. The ROC AUC was calculated as a performance matrix using the statistical package *pROC* in R^38^.

### Statistical analysis

Within each cohort, group means were compared using ANOVA, followed by Tukey’s HSD post-hoc multiple comparisons to control the type I error. To determine whether a statistically significant association existed between two categorical variables, Fisher’s exact test was performed. Metabolite concentrations were presented as medians with interquartile ranges (IQR). Between cohorts, the mean concentrations of the four metabolites and eRF_fel_ in healthy non-CKD cats were compared using the Welch’s t-test. Spearman’s correlation analysis was performed to examine the associations between eRF_fel_, age, body weight, and key renal markers SDMA, creatinine, and USG. Data analysis and visualization were performed in statistical software R (version 4.4.1) and GraphPad Prism (version 10.4.0).

## Supplementary information

Supplemental Files

## Data Availability

The datasets generated and analyzed during the current study are not publicly available due to pet privacy concerns but are available from the corresponding author on reasonable request.
