# Supplementary material for: Evaluation of equation as a classifier for kidney function in domestic cats: a proof-of-concept study
Source: NPJ Aging. 2026 May 25;12(1):128. doi: 10.1038/s41514-026-00417-2 (PMC13578686; doi:10.1038/s41514-026-00417-2)
Supplement: Supplementary file 1 [file 41514_2026_417_MOESM1_ESM.pdf]

Supplemental Figures

Fig. S1

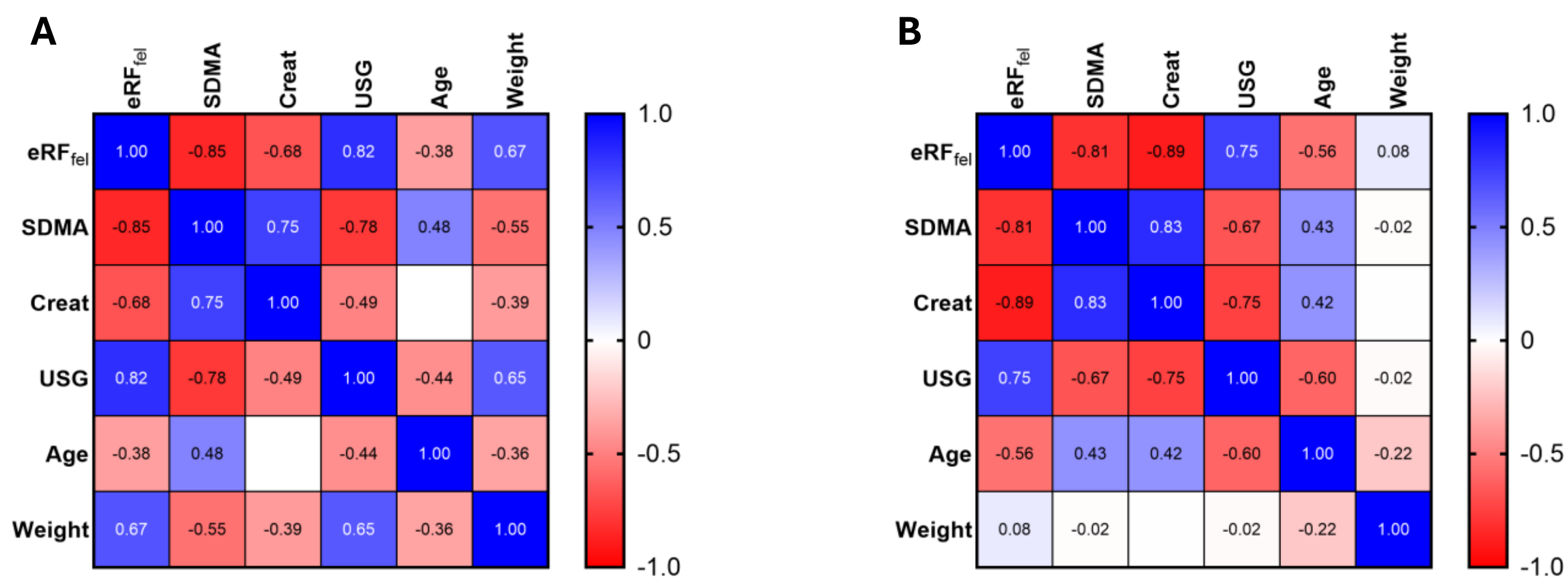

Fig. S2

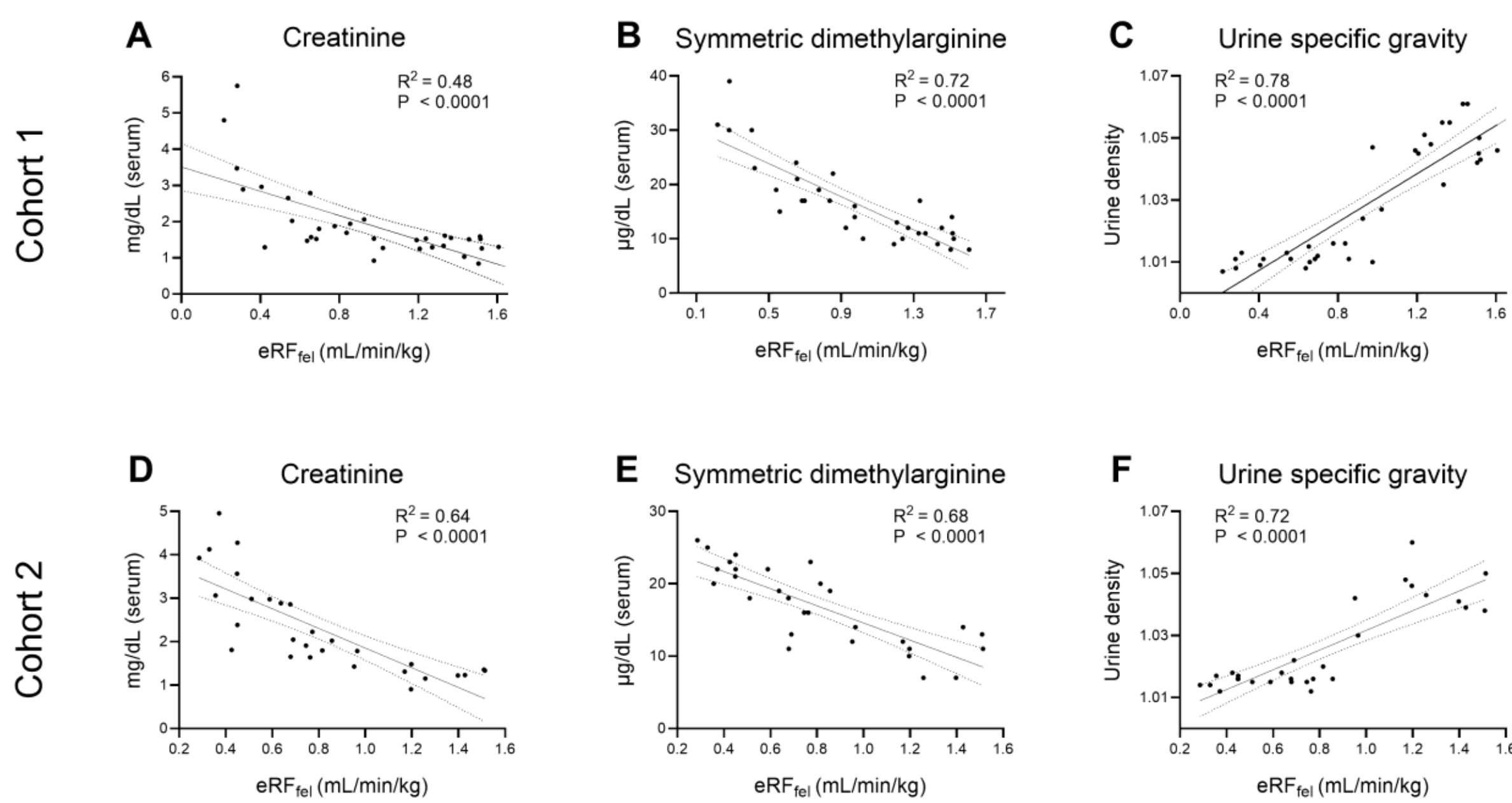

Figure Legends

Fig. S1. Association analyses between eRF_{fel} and physical, biochemical and clinical features of the cats. Spearman's rank correlation in (**A**) cohort 1 and (**B**) cohort 2. Blue color represents positive correlations while red for negative ones. The numbers inside squares indicate Pearson's correlation coefficient (ρ). Creat, serum creatinine; SDMA, symmetric dimethylarginine; USG, urine specific gravity; eRF_{fel}, estimated feline renal function.

Fig. S2. Simple linear regression analyses of serum creatinine, serum symmetric dimethylarginine (SDMA), urine specific gravity (USG) (**A-C**) on eRFfel, respectively, in cohort 1 and (**D-F**) in cohort 2. R^2, coefficient of determination, measures the goodness of fit for a regression model, while the P-value tests the null hypothesis that the slope is zero. The 95% confidence intervals of the estimated regression line were generated. The eRF_{fel}, estimated feline renal function.

Supplemental Tables

Table S1. Comparison of four metabolites and eRF_{fel} of healthy cats between the two cohorts

	Mean		Median		P value
	cohort 1	cohort 2	cohort 1	cohort 2	
eRF_{fel}	1.37	1.29	1.4	1.26	0.33
Psi	0.72	0.77	0.72	0.79	0.24
AcThr	0.07	0.07	0.07	0.07	0.09
PAGln	0.01	0.01	0.01	0.01	0.72
Trp	11.15	10.6	11.1	10.05	0.64

* Only healthy cats of cohort 1 (n=14) and cohort 2 (n=9) were included
** P value from Welch's t-test

Table S2. Multiple reaction monitoring (MRM) transitions, retention time (RT), dwell time (dwell), cone energy (cone) and collision energy (CE)

	Precursor (m/z)	Product (m/z)	RT (min)	Dwell (s)	Cone (V)	CE (V)
acetylthreonine	162.1	56	1.22	0.038	20	17
acetylthreonine	162.1	74	1.22	0.038	20	15
acetylthreonine*	162.1	120	1.22	0.038	20	8
N-acetyl-d3-L-threonine-2,3-d2	167.1	58	1.22	0.038	20	17
N-acetyl-d3-L-threonine-2,3-d2	167.1	77	1.22	0.038	20	15
N-acetyl-d3-L-threonine-2,3-d2*	167.1	123	1.22	0.038	20	8
tryptophan*	205.1	146	1.60	0.021	20	30
tryptophan	205.1	188	1.60	0.021	20	22
tryptophan-13C11*	216.1	155	1.60	0.021	20	17
tryptophan-13C11	216.1	199	1.60	0.021	20	10
phenylacetylglutamine	265.1	84	0.89	0.038	20	30
phenylacetylglutamine	265.1	91	0.89	0.038	20	30
Phenylacetylglutamine*	265.1	130	0.89	0.038	20	12
phenylacetylglutamine-D5	270.1	84	0.89	0.038	20	30
phenylacetylglutamine-D5	270.1	96	0.89	0.038	20	30
phenylacetylglutamine-D5*	270.1	130	0.89	0.038	20	12
pseudouridine	245.1	125	1.56	0.021	15	17
pseudouridine	245.1	155	1.56	0.021	15	15
Pseudouridine*	245.1	191	1.56	0.021	15	15
pseudouridine-13C,15N2	248.1	128	1.56	0.021	15	17
pseudouridine-13C,15N2	248.1	158	1.56	0.021	15	15
pseudouridine-13C,15N2	248.1	194	1.56	0.021	15	15

*Denotes the transition used as quantifier
